# EGFR is not a major driver for osteosarcoma cell growth in vitro but contributes to starvation and chemotherapy resistance

**DOI:** 10.1186/s13046-015-0251-5

**Published:** 2015-11-02

**Authors:** Florian Sevelda, Lisa Mayr, Bernd Kubista, Daniela Lötsch, Sushilla van Schoonhoven, Reinhard Windhager, Christine Pirker, Michael Micksche, Walter Berger

**Affiliations:** Department of Orthopaedics, Medical University of Vienna, Waehringer Guertel 18-20, 1090 Vienna, Austria; Institute of Cancer Research and Comprehensive Cancer Center, Department of Medicine I, Medical University Vienna, Borschkegasse 8a, 1090 Vienna, Austria

**Keywords:** Osteosarcoma, Epidermal growth factor receptor, EGFR, Gefitinib, Therapy resistance

## Abstract

**Background:**

Enhanced signalling via the epidermal growth factor receptor (EGFR) is a hallmark of multiple human carcinomas. However, in recent years data have accumulated that EGFR might also be hyperactivated in human sarcomas. Aim of this study was to investigate the influence of EGFR inhibition on cell viability and its interaction with chemotherapy response in osteosarcoma cell lines.

**Methods:**

We have investigated a panel of human osteosarcoma cell lines regarding EGFR expression and downstream signalling. To test its potential applicability as therapeutic target, inhibition of EGFR by gefitinib was combined with osteosarcoma chemotherapeutics and cell viability, migration, and cell death assays were performed.

**Results:**

Osteosarcoma cells expressed distinctly differing levels of functional EGFR reaching in some cases high amounts. Functionality of EGFR in osteosarcoma cells was proven by EGF-mediated activation of both MAPK and PI3K/AKT pathway (determined by phosphorylation of ERK1/2, AKT, S6, and GSK3β). The EGFR-specific inhibitor gefitinib blocked EGF-mediated downstream signal activation. At standard in vitro culture conditions, clinically achievable gefitinib doses demonstrated only limited cytotoxic activity, however, significantly reduced long-term colony formation and cell migration. In contrast, under serum-starvation conditions active gefitinib doses were distinctly reduced while EGF promoted starvation survival. Importantly, gefitinib significantly supported the anti-osteosarcoma activities of doxorubicin and methotrexate regarding cell survival and migratory potential.

**Conclusion:**

Our data suggest that EGFR is not a major driver for osteosarcoma cell growth but contributes to starvation- and chemotherapy-induced stress survival. Consequently, combination approaches including EGFR inhibitors should be evaluated for treatment of high-grade osteosarcoma patients.

**Electronic supplementary material:**

The online version of this article (doi:10.1186/s13046-015-0251-5) contains supplementary material, which is available to authorized users.

## Background

Osteosarcoma is the most common primary malignant bone tumor with a peak incidence in childhood and adolescence frequently occurring at sites of rapid bone growth. The long-term survival of patients with osteosarcoma has improved from 10 to 20 % to nearly 80 % within the last 25 years, due to the use of neoadjuvant chemotherapy [1]. However, this plateau has not changed for more than 15 years [2]. For patients with metastatic disease, the outcomes are distinctly worse, with less than 30 % survival at 5 years [3]. Furthermore some patients do not respond to chemotherapy and others exhibit features of multidrug resistance (MDR), probably due to overexpression of P-glycoprotein (ABCB1) [4]. Consequently, there is still an urgent demand for new and more effective therapeutic strategies.

The erbB family receptor tyrosine kinases play an important role in the control of cell cycle, proliferation, and migration of normal and cancer cells. Expression of erbB molecules, especially the epidermal growth factor receptor (EGFR, erbB1), has been described in osteosarcomas [5]. This observation opens the possibility that EGFR might be a feasible therapeutic target in osteosarcoma although activating EGFR mutations are widely missing in this aggressive primary bone tumor [5, 6]. EGFR-targeted therapies (monoclonal antibodies or small molecule TK inhibitors) have been already clinically approved for several tumors including colorectal, non-small cell lung, head and neck, as well as pancreatic cancer [7–9]. Gefitinib is a selective EGFR tyrosine kinase inhibitor currently utilized for the treatment of patients with non-small cell lung cancer harbouring activating EGFR mutations [10–12]. In the present study, we investigated the impact of EGFR inhibition on osteosarcoma cell behaviour and its interaction with chemotherapy response.

## Methods

### Reagents

Gefitinib (ZD1839, Iressa®, 4-quinazolinamine, N-(3-chloro-4-flurophenyl)-7-methoxy-6-(3-(4-morpholinyl) propoxy), a selective EGFR inhibitor, was kindly provided by AstraZeneca, and dissolved in sterile dimethyl sulphoxide (DMSO). Solutions were freshly prepared before use. As standard chemotherapeutic agents in osteosarcoma, doxorubicin, methotrexate (MTX) and cisplatin were used (Sigma, Vienna, Austria). Stock solutions were prepared for gefitinib at 10 mM in DMSO, doxorubicin at 3.5 mM in saline, cisplatin at 4 mM in dimethylformamide and MTX in a minimal amount of 1 M NaOH followed by saline to 200 mM.

### Cell cultures

MG-63, HOS, Saos-2 and U-2 OS osteosarcoma cell lines were obtained from the American Type Culture Collection (Manassas, VA). Primary osteosarcoma cell lines OS-10, OS-9, SARG and IOR-MOS were generously supplied by Dr. K. Scotlandi from the Instituti Ortopedici Rizzoli (Bologna, Italy) [13, 14]. The HL-NG cell line was established from a fibroblastic osteosarcoma surgery specimen at the Institute of Cancer Research, Vienna. Calu-3, an EGFR-overexpressing non-small cell lung cancer cell line used as positive control, was obtained from ATCC. Histological subtype of the original tumors and culture media are indicated in Additional file 1: Table S1. Cells were cultured in growth media supplemented with 10 % fetal calf serum (FCS) at 37 °C in a 5 % CO_2_ incubator. The cell lines were authenticated in all cases by array comparative genomic hybridization (Agilent, 44 k human whole genome DNA arrays) as published [15] and/or short tandem repeat (STR) fingerprinting before the start of this study.

### Cell growth and viability assays

Cells were plated (2x10^4^ cells/mL) in 100 μL per well in 96-well plates and allowed to attach for 24 h. Drugs were added in another 100 μL growth medium and cells exposed for 72 h. The proportion of viable cells was determined by 3-(4,5-dimethylthiazol-2-yl)-2,5-diphenyltetrazolium assay (MTT) following the manufacturer’s recommendations (EZ4U, Biomedica, Vienna, Austria). Cytotoxicity was expressed as IC_50_ values calculated from full dose–response curves. The interaction between the activities of combined drugs is expressed by the combination index (CI) as published by Heffeter et al. [16] using CalcuSyn software (Biosoft, Ferguson, MO). CI < 0.9, CI = 0.9–1.2 or CI >1.2 represent synergism, additive effects and antagonism, respectively.

### Apoptosis and cell cycle analyses

Induction of cell death was followed by staining of living osteosarcoma cell cultures under different treatment and serum conditions with Hoechst 33258 and propidium iodide (PI) as published [17]. Hoechst dye enters living cells and allows detection of chromatin condensation due to either mitosis or apoptosis. In contrast, PI only stains dead cells due to necrosis or apoptosis. Assays were performed in 24-well plates in duplicate and 4 optical fields per well were evaluated after 24 h by microscopic counting of apoptotic cells. Cell cycle distribution was analysed after 24 h exposure to gefitinib by PI staining of ethanol-fixed cells followed by FACS analysis as published previously [17, 18].

### Clonogenic assay

10^3^ cells per well were seeded into six-well plates. Following 24 h recovery, cells were treated with MTX, doxorubicin, cisplatin and gefitinib. At day 7 of exposure, cells were washed twice with PBS, fixed with methanol at −20 °C and stained with crystal violet. The number of colonies containing at least 100 single cells was determined by counting microscopically using a Leica DMIL (Leica, Solms, Germany).

### Protein isolation and Western blotting

Total protein and membrane protein-enriched fractions were extracted and processed for Western blotting as described [19, 20] using the following primary antibodies: pEGFR(Tyr1068), EGFR, phospho-S6 ribosomal protein (Ser240/244), S6, phospho-p44/42 MAP kinase ERK (Thr202/Tyr204), p44/42 MAP kinase ERK, pAKT (Ser473), AKT, pGSK3β (Ser9), GSK3β, (all polyclonal rabbit contained in the respective sampler kits from Cell Signaling Technology, Beverly, MA), *ß*-actin monoclonal mouse AC-15 (Sigma).

### RNA isolation

Total RNA was isolated with Trizol reagent according to standard protocols. RNA quantity and quality was determined by Nanodrop measurements (Nanodrop 1000, Thermo Fisher Scientific, Wilmington, DE). Quantity ranged between 150 and 400 ng/μl. All samples had a 260/280 ratio > 1.8.

### Real-time PCR

1000 ng of total RNA were reverse transcribed into cDNA. For real-time PCR 10 ng were used for each amplification reaction (performed in triplicate). Real-time polymerase chain reaction (PCR) was performed as described [21]. Expression levels of ABCB1 mRNA levels were determined using the Maxima SYBR Green/ROX qPCR Mastermix (Thermo Fisher Scientific) with β-actin serving as a reference gene. ABCB1 primer sequence [22, 23]: ABCB1 sense: 5’-CCCATCATTGCAATAGCAGG-3’ and ABCB1 antisense: 5’-GTTCAAACTTCTGCTCCTGA-3’. β-actin primer sequence: β-actin sense: 5’-GGATGCAGAAGGAGATCACTG-3’ and β-actin antisense: 5’-CGATCCACACGGAGTACTTG-3’. For determination of EGFR mRNA expression Taqman assays using Taqman Maxima Probe/ROX qPCR Mastermix (Thermo Fisher Scientific) were performed. Taqman probes for EGFR (Hs01076078_m1) and GAPDH (Hs99999905_m1) were purchased from (Applied Biosystems, Waltham, MA). Quantification of ABCB1 and EGFR mRNA expression was calculated by the comparative Ct method using β-actin and GAPDH as reference genes, respectively. Experiments were performed twice delivering comparable results.

### Migration assays

In wound healing assays, scratches were applied to confluent cultures in six-well plates (tissue culture treated, CytoOne® Starlab, Hamburg, Germany) using a pipette tip. Medium was renewed and the indicated treatments were added. Scratches were photographed after 0, 24 and 48 h and wound closure was calculated from the micrographs with T-Scratch (Computational Science & Engineering Laboratory, ETH Zurich, Switzerland). After a migration period of 24 h and 48 h, migrated cells on the bottom of the filter (cell culture insert for 24-well plates, 8.0 μm pore size, Falcon™ ThermoFisher Scientific) were counted (4 optical fields per well). Additionally, the bottom wells were further cultivated for 7 more days, fixed with methanol, stained with crystal violet and cell clones counted microscopically.

### Statistical analysis

Statistical analysis was performed using GraphPad Prism 5.0 software. All data are expressed as mean ± S.D. Statistical significance of differences was analysed by using unpaired Student ´s *t*-test and one-or two-way ANOVA as appropriate followed by Bonferroni post-tests. A *p*-value < 0.05 was considered statistically significant. Throughout the study the following classification is used: *, *p* < 0.05; **, *p* < 0.01 ***, *p* < 0.001.

## Results

### Osteosarcoma cells express functional EGFR

**Fig. 1:**
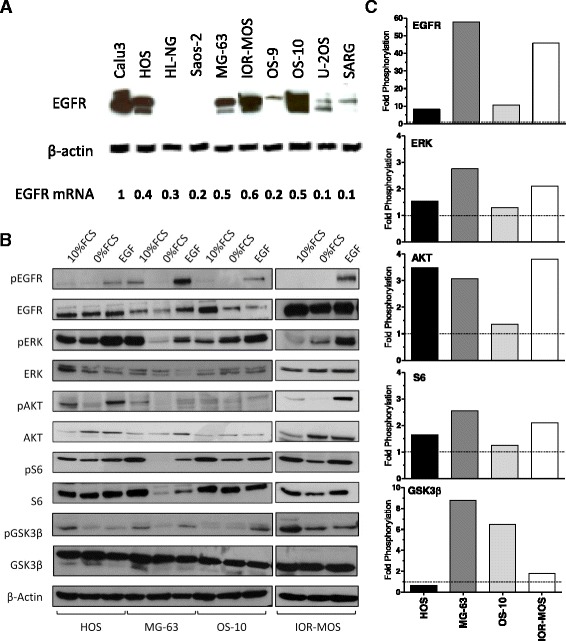
Expression and functionality of EGFR in osteosarcoma cell lines. **a** Western blot analysis of EGFR in 9 membrane protein-enriched fractions prepared from human osteosarcoma cell lines is opposed to an extract from Calu 3, a gefitinib-sensitive NSCLC cell line, used as a positive control. β-actin is shown as loading control. EGFR mRNA expression levels are indicated relatively to Calu 3 set as 1. **b** Four representative osteosarcoma cell lines with enhanced EGFR expression were either cultured under FCS at 10 % (*10 % FCS*), serum-starved for 24 h (*0 % FCS*) or stimulated with EGF (50 ng/ml for 15 min) following serum starvation for 24 h (*EGF*). The impact on EGFR phosphorylation and downstream activation of MAPK (ERK) and PI3K (AKT, S6, GSK3β) signaling pathways was determined by Western blot analysis. **c** Bar charts depict densitometric quantification of the Western blots (ImageJ Software) from (**b**) and data are given as phosphorylation of the indicated proteins relative to serum-starved conditions (*0 % FCS*)

**Fig. 2:**
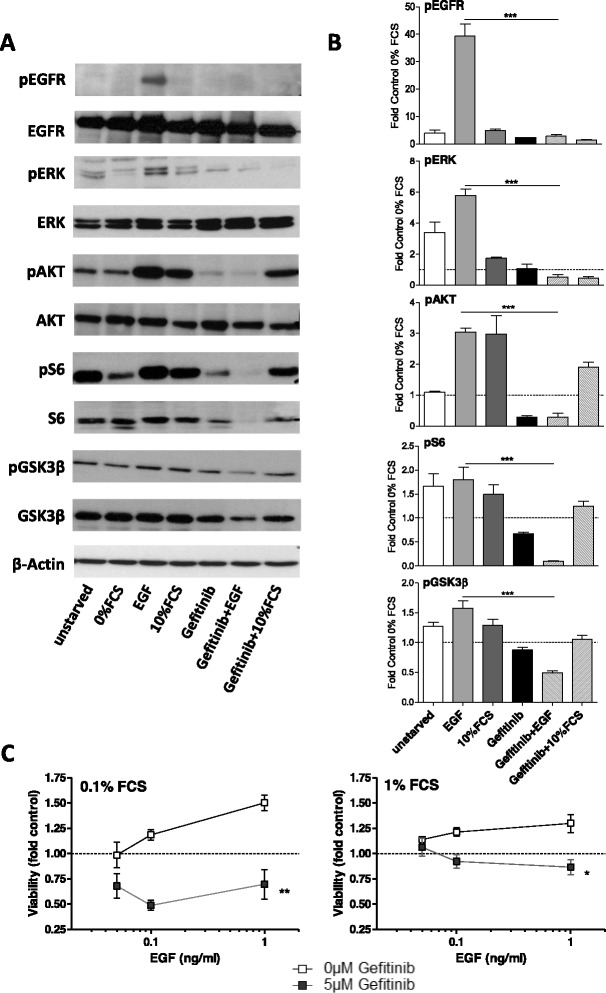
Impact of EGF and EGFR inhibition by gefitinib on signaling pathway activation and starvation survival of osteosarcoma cells. **a** The impact of gefitinib (10 μM, 30 min) as indicated on phosphorylation of EGFR, ERK, S6, AKT and GSK3β in IOR-MOS cells either cultured under 10 % FCS (*unstarved*), serum-starved for 24 h (*0 % FCS*) or stimulated after serum starvation with EGF (50 ng/ml, 15 min; *EGF*) or 10 % FCS (*10 % FCS*) was determined by Western blot analysis. **b** Densitometric quantification of Western blots (ImageJ Software) from three experiments (one representative shown under *a*) for the indicated signal proteins. Data are given relative to the phosphorylation levels at serum-starved conditions set as 1. **c** Viability of IOR-MOS cells was determined by MTT assays after 72 h serum starvation (1 % or 0.1 % FCS as indicated) under increasing EGF concentrations without or with gefitinib (5 μM). Significance of the gefitinib impact: two-way ANOVA with Bonferroni’s post hoc test; * *p* < 0.05; ** *p* < 0.01

EGFR was readily detectable in membrane protein-enriched fractions from 7/9 cell models with HOS, IOR-MOS and OS-10 cells showing high expression even comparable to the EGFR-driven lung cancer cell line Calu-3 (Fig. 1a). Expression in HL-NG and Saos-2 cells was low and only visible after extended exposure time in Western blot analysis (Additional file 2: Figure S1). The respective EGFR mRNA was detectable by real-time PCR in all osteosarcoma cell lines (Fig. 1a) and correlated roughly with membrane-residing EGFR levels. EGFR gene expression levels are opposed to histological subtype and the well known predictive osteosarcoma biomarker ABCB1 (P-glycoprotein) [4] in Additional file 1: Table S1. In order to determine functionality of EGFR in osteosarcoma cells we selected four cell lines (HOS, MG-63, OS-10, IOR-MOS) with high EGFR expression for signal transduction analysis. Application of EGF for 15 min to serum-starved osteosarcoma cells led in most cases to a distinct activation of EGFR phosphorylation and of the respective downstream MAPK (ERK phosphorylation) and PI3K/AKT (AKT, S6, and GSK3β phosphorylation) signaling pathways (Fig. 1b, densitometric quantification in Fig. 1c). EGFR activation restored signal transduction in the majority of the tested cell models to the level of unstarved cells (10 % FCS) or even distinctly higher. Application of gefitinib significantly blocked EGF-mediated activation of the PI3K/AKT and ERK pathways even below the serum-starved control (IOR-MOS cells are shown representatively as a highly EGF-responsive cell model in Fig. 2a, b). In contrast, pathway activation by FCS was comparably insensitive against EGFR inhibition. Interestingly, residual phosphorylation of AKT and S6 under serum-starved conditions was further reduced by gefitinib.

### Osteosarcoma cells are comparably insensitive to EGFR blockade at standard culture conditions but sensitive at serum starvation

At standard culture conditions with 10 % FCS, treatment with increasing concentrations of gefitinib for 72 h caused a dose-dependent decrease in the number of viable cells in all tested osteosarcoma cell lines with significantly differing sensitivities (Table 1). However, all IC_50_ values were generally above 10 μM thus distinctly exceeding clinical achievable doses [24]. In contrast, IC_50_ values for gefitinib dramatically dropped at serum-reduced starvation conditions (0.1 and 1 % FCS) in all osteosarcoma cell lines except U-2 OS. Accordingly, application of EGF in an appropriate dose range (which differed between cell lines) protected osteosarcoma cells against serum starvation independent of the endogenous EGFR expression level (examples for cell lines with high endogenous EGFR expression in Fig. 2c and Additional file 3: Figure S2A and with comparable low expression levels in Additional file 3: Figure S2B). A comparable growth stimulating effect of EGF was not detectable at standard culture conditions (data not shown). Gefitinib attenuated or blocked this protective effect and suppressed viability in some cases even below the serum-starved control. Solely viability of the highly EGFR positive cell line OS-10 could not be enhanced by application of exogenous EGF despite high gefitinib responsiveness under serum-starved conditions (data not shown, compare Table 1). The anti-osteosarcoma effect of gefitinib was based on enhanced apoptosis induction at lower serum concentrations (three osteosarcoma cell lines with different EGFR expression levels are shown in Additional file 4: Figure S3). Additionally, surviving cells accumulated either in G0/G1 or S cell cycle phase depending on the cell line tested (Additional file 5: Figure S4).

**Table 1:** Anticancer activity of gefitinib as a single agent against 9 osteosarcoma cell lines

	Gefitinib (IC_50_; μM)^a^		
	FCS^b^		
Cell line	0.1 %	1 %	10 %
HL-NG	6.9	6.1	9.2
MG-63	5.1	15.3	16.3
HOS	12.1	18.2	19.9
Saos-2	13.8	21.5	27.1
OS-10	6.0	10.1	32.8
OS-9	7.3	8.5	29.5
SARG	3.6	4.1	31.2
IOR-MOS	2.8	10.4	31.5
U-2 OS	28.1	35.9	36.0

^a^Gefitinib concentrations causing a reduction of cell viability by 50 % as compared to the untreated controls

^b^Amount of fetal calf serum (FCS) added to the respective growth media

### Impact of EGFR blockade on osteosarcoma cell migration

**Fig. 3:**
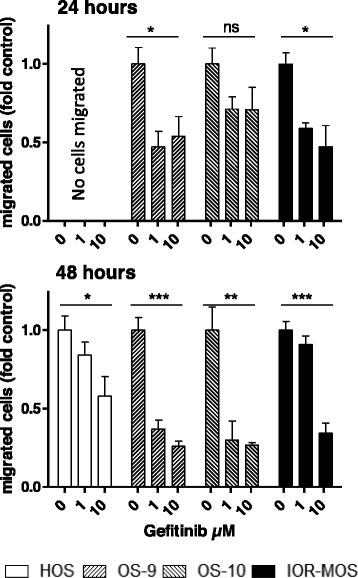
EGF/EGFR-mediated signals contribute to the migratory potential of osteosarcoma cells. Transwell migration assays (two experiments in duplicate) were performed for 24 h and 48 h in growth medium with 1 % FCS without and with addition of gefitinib as indicated. Migrated cells at the lower side of the membrane were counted microscopically in 4 optical fields/membrane in duplicate and normalized to the untreated control. One-way ANOVA with Bonferroni’s post hoc test; * *p* < 0.05; ** *p* < 0.01; *** *p* < 0.001

Next, the effect of EGFR blockade by gefitinib on osteosarcoma cell migration was determined using trans-well assays (Fig. 3). The indicated osteosarcoma cells exhibited significantly reduced migratory potential following EGFR inhibition leading to elongated time frames of transmembrane passage.

### Gefitinib sensitizes osteosarcoma cells against chemotherapy

**Fig. 4:**
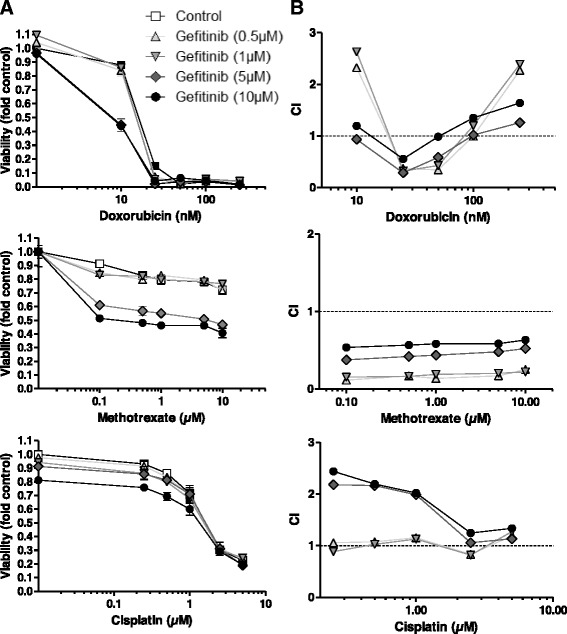
Gefitinib sensitizes osteosarcoma cells against chemotherapy. **a** The effect of EGFR blockade by gefitinib at the indicated concentrations on responsiveness to the standard osteosarcoma therapeutics doxorubicin, MTX and cisplatin was tested by MTT-based survival assays at 72 h exposure time in triplicate. Representatively one out of three experiments for OS-10 cells is shown. **b** Combination Index (=CI) based on the data under (**a**) were calculated as published. CI values < 0.9 indicates synergistic effects, CI = 0.9-1.2 additive and CI > 1.2 antagonistic effects

**Fig. 5:**
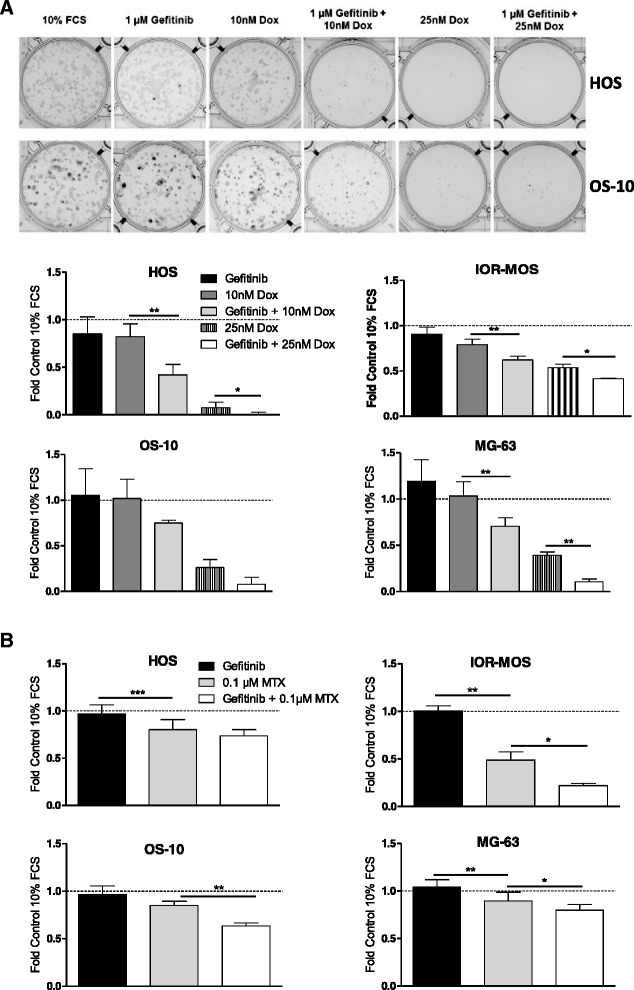
EGFR contributes to chemotherapy resistance of osteosarcoma cells. **a** Effects of combined long-term application of doxorubicin (Dox) and gefitinib were tested by clonogenic survival assays. Representative wells with crystal violet-stained osteosarcoma cell clones (HOS, OS-10) are opposed to the densitometric quantification from two experiments in triplicate. **b** Data for the combined application of MTX and gefitinib in the long-term exposure were analysed as described under (**a**). Students *t*-test; * *p* < 0.05; ** *p* < 0.01; *** *p* < 0.001

**Fig. 6:**
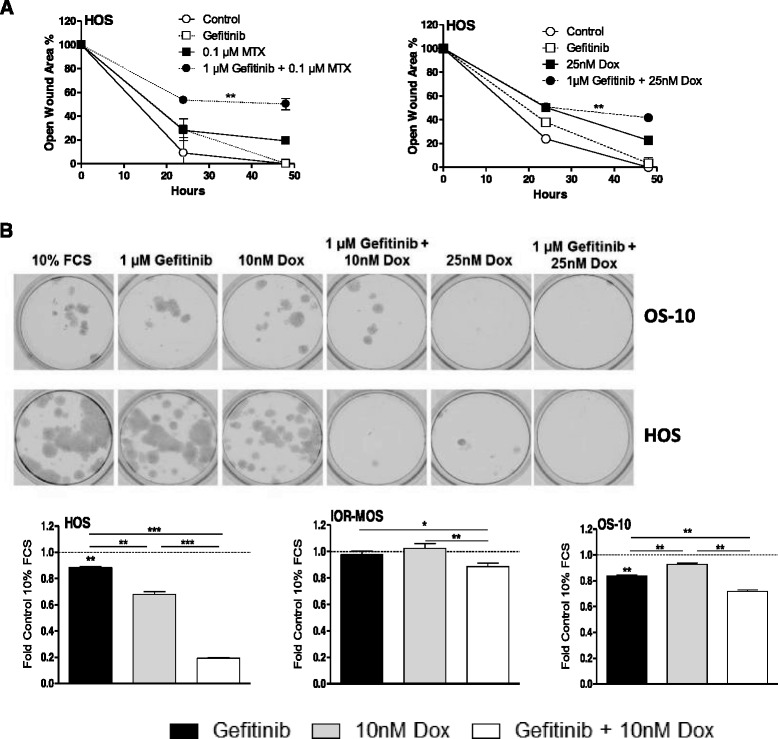
EGFR blockade synergizes with standard chemotherapy to inhibit the migratory potential of osteosarcoma cells. **a** For scratch assays, HOS cells were treated with standard chemotherapy (MTX, doxorubicin) without and with 1 μM gefitinib and wound healing was followed at two positions of the wound up to 48 h as indicated. Means and S.D. of two experiments are shown. **b** Transwell migration assays were performed with HOS, IOR-MOS and OS-10 cells for 48 h at subtoxic concentrations of doxorubicin without and with 1 μM gefitinib. Clonogenic growth in the lower chamber was analysed as under Fig. 3b. Selected wells containing crystal violet-stained osteosarcoma cell clones are opposed to quantification of experiments performed in triplicate. One-way ANOVA with Bonferroni’s post hoc test (**a**) and Students *t*-test (**b**); * *p* < 0.05; ** *p* < 0.01; *** *p* < 0.001

The impact of a combined application of the EGFR inhibitor gefitinib with standard chemotherapy used in osteosarcoma cells (e.g. doxorubicin, MTX, cisplatin) was tested for short- and long-term exposure by MTT (72 h) and clonogenic (7 days) assays, respectively. In the MTT assay gefitinib moderately but significantly synergized with doxorubicin in most (U-2 OS, MG-63, HOS, Saos-2, OS-9 and OS-10) and MTX in several (Saos-2, OS-9 and OS-10) osteosarcoma cell lines (growth curves and combination indices representatively shown for OS-10 cells in Fig. 4). In contrast, EGFR inhibition generally antagonized the effect of cisplatin (Fig. 4). The sensitizing effect against doxorubicin and MTX was markedly stronger in the long-term exposure experiments and observable already at 1 μM gefitinib in all tested cell lines (Fig. 5a and b). With regard to the migratory potential, combined application of gefitinib and standard chemotherapy further decreased migration in wound healing analyses (HOS cells are shown representatively in Fig. 6a). Clonogenic growth at the lower chamber in trans-well migration assay (48 h migration) was determined in several osteosarcoma cell lines (Fig. 6b). Combined application of gefitinib and doxorubicin resulted in synergistically decreased migration/clonogenic potential compared to both doxorubicin and gefitinib as single agents in three of four cell lines (HOS, IOR-MOS and OS-10), whereas MG-63 cells remained unaffected (data not shown).

## Discussion

In accordance with previous studies on human osteosarcoma cell lines and tissues [6, 25], we found frequent and profound expression of EGFR in osteosarcoma cells suggesting that it might be an attractive therapy target. However, the actual functional contribution of EGFR to osteosarcoma cell biology has remained widely unexplored and literature regarding its prognostic value is contradictory. Some authors did not find a relation between EGFR expression and prognosis or treatment outcome [6, 26]. In contrast, high EGFR expression was associated with significantly shorter survival times and disease free intervals in canine osteosarcomas [27]. Furthermore, Wen et al. found increased EGFR levels in metastases and local recurrences when compared with primary tumors [5]. Surprisingly, better prognosis was reported for patients with higher EGFR expression by Kersting et al. [28] A shorter allele length of a CA repeating sequence in the intron I of the EGFR gene tended to be associated with increased EGFR expression. However there was no correlation between allele length and neoadjuvant chemotherapy response or long-term clinical outcome [29]. Additionally, targeting of EGFR by cetuximab was suggested to induce osteosarcoma growth inhibition via antibody-dependent tumor cell phagocytosis by a M2-like macrophage subpopulation [30].

In our study we could demonstrate functionality of the EGFR in osteosarcoma cell lines leading to EGF-mediated activation of the MAPK and PI3K/AKT pathways. Moreover, blockade of EGFR by gefitinib inhibited osteosarcoma cell proliferation at standard culture conditions only at comparably high concentrations not likely to be reached in vivo. This suggests that EGFR is not a central driver of osteosarcoma cell proliferation under in vitro cell culture conditions. Accordingly, gefitinib up to 20 μM did not reduce viability of several osteosarcoma cell lines in a previous study [6]. However, under reduced serum concentrations, mimicking the starvation situation inside tumor nodules in vivo, the IC_50_ values for gefitinib distinctly dropped. Also in other cellular stress conditions - including very sparse seeding in clonogenic assays and chemotherapy-induced cytotoxicity - EGFR-mediated signals supported osteosarcoma cell survival. At serum-reduced conditions, EGFR inhibition by gefitinib induced marked apoptotic osteosarcoma cell death. Additionally, gefitinib led to accumulation of cells in either G0/G1 or S phase of the cell cycle. Comparable observations have been published as a consequence of imatinib mesylate exposure of osteosarcoma cells. Interestingly, the authors suggested EGFR as one important target inhibited by this clinically approved kinase inhibitor [31]. Moreover, osteosarcoma cell migration was significantly reduced by EGFR blockade and this effect was synergistically enhanced in combination with chemotherapy. These data are in accordance with a previous study demonstrating that selective in vitro inhibition of EGFR induced decreased motility, colony formation and invasiveness of osteosarcoma cells [32]. The non-selective pan-erbB inhibitor CI-1033 achieved IC_50_ values at concentrations of approximately 1 μM after 4 days of drug exposure [33]. Pahl et al. showed cytotoxic effect of the anti-EGFR monoclonal antibody cetuximab induced by natural killer cell activation and following lysis of osteosarcoma cells [34]. In both latter studies cytotoxic activity was not correlated with the level of EGFR expression. This is similar to our observations that neither the growth-inhibitory nor cell death-inducing effects of gefitinib were depending on the level of endogenous EGFR expression. Additionally, EGFR levels did not correlate with the histology of the original tumor. Interestingly exogenous EGF upregulated viability of osteosarcoma cells in the majority but not all cell lines tested. Thus, the highly EGFR-positive and gefitinib-sensitive OS-10 cell line was surprisingly not responsive to recombinant EGF under serum starvation. This suggests that EGFR might be stimulated even under serum-starved conditions by endogenous ligands in this cell model. Correspondingly, gefitinib reduced residual PI3K/AKT pathway activity determined as phosphorylation of AKT, S6 and GSK3β under serum-starved conditions. This strongly suggests that autocrine stabilisation of the PI3K/AKT pathway via EGFR supports starvation resistance of osteosarcoma cells as also observed in glioblastoma cells [20]. Accordingly, activation of PI3K/AKT pathway and osteosarcoma cell migration was recently demonstrated by endogenous expression of transforming growth factor α via binding to EGFR [35].

In many clinical treatment schemes, combination approaches of oncogenic kinase inhibitors and cytotoxic drugs turned out to be highly successful [36–38]. Thus, we investigated combination of EGFR inhibition by gefitinib with osteosarcoma standard chemotherapeutics. We detected distinct synergism for combination with doxorubicin and MTX in osteosarcoma cells. ABCB1 efflux pump expression has been suggested as a major regulator of osteosarcoma chemotherapy response [4]. As gefitinib is known to block ABCB1 efflux function [39], the observed synergism with the ABCB1 substrate doxorubicin might be based rather on enhanced drug accumulation as blockade of an EGFR-mediated survival function. However, ABCB1 gene expression detected by real-time PCR was comparably low in all osteosarcoma cell lines making this assumption unlikely. Accordingly, synergism was also found for the none ABCB1 substrate MTX [40], while the combination with cisplatin, also not transported by ABCB1, was widely antagonistic. Several previous preclinical studies on other tumor entities have shown additive and super-additive but also antagonistic interactions for EGFR inhibitors and chemotherapy [41, 42]. Repeatedly, studies on EGFR targeting combined with cytotoxic drugs have been confirmed clinically, the most convincing being the therapeutic success achieved by the cetuximab-irinotecan combination in colorectal cancer [43]. Combination of gefitinib with doxorubicin in soft tissue sarcomas showed synergistic impacts on cell proliferation and apoptosis induction compared to single agents. Furthermore, in animal models for soft tissue sarcoma combined low-dose doxorubicin and gefitinib were markedly synergistic [44].

Two clinical phase I studies with advanced solid tumors revealed partial responses for a combination therapy of the EGFR inhibitors gefitinib and erlotinib with both cediranib (VEGFR inhibitor) and temozolomide [45, 46]. Only 3 patients with osteosarcoma were included in these studies. One of them developed a partial response, which is encouraging for further studies on drug combination including EGFR tyrosine kinase inhibitors in osteosarcoma.

## Conclusions

EGFR-mediated survival signals protect human osteosarcoma cells against cellular stress conditions including several antineoplastic drugs. Consequently, combination approaches of EGFR inhibitors in addition to chemotherapy should be evaluated for treatment of high-grade osteosarcoma patients.

## Additional files

Additional file 1: Table S1. Phenotypic characteristics as well as EGFR and ABCB1 gene expression of the investigated osteosarcoma cell models. (DOCX 14 kb) Additional file 2: Figure S1. EGFR expression in osteosarcoma cells. For lowly EGFR positive osteosarcoma cell lines in Fig. 1a Western blots of membrane-enriched fractions were prepared and subjected to extended film exposure. (PDF 26 kb) Additional file 3: Figure S2. Impact of EGF and EGFR inhibition on starvation survival of osteosarcoma cells with comparably high (A) and low (B) EGFR expression levels. Viability of osteosarcoma cells was determined by MTT assays after 72 h serum starvation (1 % or 0.1 % FCS) under increasing EGF concentrations without or with gefitinib (5 μM) as indicated. Significance of the gefitinib impact: ** p < 0.01; *** p < 0.001 by Two-way ANOVA with Bonferroni’s post hoc test. (PDF 71 kb) Additional file 4: Figure S3. Impact of serum starvation on apoptosis induction by gefitinib in osteosarcoma cells. The indicated osteosarcoma cell lines were treated with gefitinib (1 μM and 10 μM) for 24 h in 1 and 10 % FCS containing culture medium. Hoechst 33258 and propidium iodide were added for 4 h and photomicrographs of 4 optical fields per well were evaluated for cells with condensed chromatin indicating apoptosis execution. * *p* < 0.05; ** *p* < 0.01; *** *p* < 0.001 by One-way ANOVA with Bonferroni’s post hoc test. (PDF 62 kb) Additional file 5: Figure S4. Impact of EGFR inhibition by gefitinib on cell cycle distribution. The indicated osteosarcoma cell lines were treated with gefitinib (1 μM, 5 μM and 10 μM) for 24 h in 1 % FCS containing culture medium and opposed to untreated cells at 10 % FCS (control). Cell cycle distribution was analysed by PI staining followed by FACS analysis. (PDF 56 kb)

## Abbreviations

CI: Combination index
DMSO: Dimethyl sulfoxide
EGFR: Epidermal growth factor receptor
FCS: Fetal calf serum
MDR: Multidrug resistance
MTX: Methotrexate
PI: Propidium iodide
STR: Short tandem repeat
TK: Tyrosine kinase

## References

[1] Marina N, Gebhardt M, Teot L, Gorlick R (2004). Biology and therapeutic advances for pediatric osteosarcoma. Oncologist.

[2] Bacci G, Ferrari S, Longhi A, Perin S, Forni C, Fabbri N (2001). Pattern of relapse in patients with osteosarcoma of the extremities treated with neoadjuvant chemotherapy. Eur J Cancer.

[3] Kager L, Zoubek A, Potschger U, Kastner U, Flege S, Kempf-Bielack B (2003). Primary metastatic osteosarcoma: presentation and outcome of patients treated on neoadjuvant Cooperative Osteosarcoma Study Group protocols. J Clin Oncol.

[4] Baldini N, Scotlandi K, Barbanti-Brodano G, Manara MC, Maurici D, Bacci G (1995). Expression of P-glycoprotein in high-grade osteosarcomas in relation to clinical outcome. N Engl J Med.

[5] Wen YH, Koeppen H, Garcia R, Chiriboga L, Tarlow BD, Peters BA (2007). Epidermal growth factor receptor in osteosarcoma: expression and mutational analysis. Hum Pathol.

[6] Lee JA, Ko Y, Kim DH, Lim JS, Kong CB, Cho WH (2012). Epidermal growth factor receptor: is it a feasible target for the treatment of osteosarcoma?. Cancer Res Treat.

[7] Manning HC, Merchant NB, Foutch AC, Virostko JM, Wyatt SK, Shah C (2008). Molecular imaging of therapeutic response to epidermal growth factor receptor blockade in colorectal cancer. Clin Cancer Res.

[8] Wakeling AE, Guy SP, Woodburn JR, Ashton SE, Curry BJ, Barker AJ (2002). ZD1839 (Iressa): an orally active inhibitor of epidermal growth factor signaling with potential for cancer therapy. Cancer Res.

[9] Moore MJ, Goldstein D, Hamm J, Figer A, Hecht JR, Gallinger S (2007). Erlotinib plus gemcitabine compared with gemcitabine alone in patients with advanced pancreatic cancer: a phase III trial of the National Cancer Institute of Canada Clinical Trials Group. J Clin Oncol.

[10] Hida T, Ogawa S, Park JC, Park JY, Shimizu J, Horio Y (2009). Gefitinib for the treatment of non-small-cell lung cancer. Expert Rev Anticancer Ther.

[11] Kitagawa D, Yokota K, Gouda M, Narumi Y, Ohmoto H, Nishiwaki E (2013). Activity-based kinase profiling of approved tyrosine kinase inhibitors. Genes Cells.

[12] Eckstein N, Roper L, Haas B, Potthast H, Hermes U, Unkrig C (2014). Clinical pharmacology of tyrosine kinase inhibitors becoming generic drugs: the regulatory perspective. J Exp Clin Cancer Res.

[13] Benini S, Baldini N, Manara MC, Chano T, Serra M, Rizzi S (1999). Redundancy of autocrine loops in human osteosarcoma cells. Int J Cancer.

[14] Mohseny AB, Machado I, Cai Y, Schaefer KL, Serra M, Hogendoorn PC (2011). Functional characterization of osteosarcoma cell lines provides representative models to study the human disease. Lab Invest.

[15] Mathieu V, Pirker C, Schmidt WM, Spiegl-Kreinecker S, Lotsch D, Heffeter P (2012). Aggressiveness of human melanoma xenograft models is promoted by aneuploidy-driven gene expression deregulation. Oncotarget.

[16] Heffeter P, Atil B, Kryeziu K, Groza D, Koellensperger G, Korner W (2013). The ruthenium compound KP1339 potentiates the anticancer activity of sorafenib in vitro and in vivo. Eur J Cancer.

[17] Dornetshuber-Fleiss R, Heffeter P, Mohr T, Hazemi P, Kryeziu K, Seger C (2013). Destruxins: fungal-derived cyclohexadepsipeptides with multifaceted anticancer and antiangiogenic activities. Biochem Pharmacol.

[18] Heffeter P, Jakupec MA, Korner W, Chiba P, Pirker C, Dornetshuber R (2007). Multidrug-resistant cancer cells are preferential targets of the new antineoplastic lanthanum compound KP772 (FFC24). Biochem Pharmacol.

[19] Steiner E, Holzmann K, Pirker C, Elbling L, Micksche M, Sutterluty H (2006). The major vault protein is responsive to and interferes with interferon-gamma-mediated STAT1 signals. J Cell Sci.

[20] Lotsch D, Steiner E, Holzmann K, Spiegl-Kreinecker S, Pirker C, Hlavaty J (2013). Major vault protein supports glioblastoma survival and migration by upregulating the EGFR/PI3K signalling axis. Oncotarget.

[21] Fischer H, Taylor N, Allerstorfer S, Grusch M, Sonvilla G, Holzmann K (2008). Fibroblast growth factor receptor-mediated signals contribute to the malignant phenotype of non-small cell lung cancer cells: therapeutic implications and synergism with epidermal growth factor receptor inhibition. Mol Cancer Ther.

[22] Miklos W, Pelivan K, Kowol CR, Pirker C, Dornetshuber-Fleiss R, Spitzwieser M (2015). Triapine-mediated ABCB1 induction via PKC induces widespread therapy unresponsiveness but is not underlying acquired triapine resistance. Cancer Lett.

[23] Noonan KE, Beck C, Holzmayer TA, Chin JE, Wunder JS, Andrulis IL (1990). Quantitative analysis of MDR1 (multidrug resistance) gene expression in human tumors by polymerase chain reaction. Proc Natl Acad Sci U S A.

[24] Rahman AF, Korashy HM, Kassem MG (2014). Gefitinib. Profiles Drug Subst Excip Relat Methodol.

[25] Hughes DP, Thomas DG, Giordano TJ, Baker LH, McDonagh KT (2004). Cell surface expression of epidermal growth factor receptor and Her-2 with nuclear expression of Her-4 in primary osteosarcoma. Cancer Res.

[26] Freeman SS, Allen SW, Ganti R, Wu J, Ma J, Su X (2008). Copy number gains in EGFR and copy number losses in PTEN are common events in osteosarcoma tumors. Cancer.

[27] Selvarajah GT, Verheije MH, Kik M, Slob A, Rottier PJ, Mol JA (2012). Expression of epidermal growth factor receptor in canine osteosarcoma: association with clinicopathological parameters and prognosis. Vet J.

[28] Kersting C, Gebert C, Agelopoulos K, Schmidt H, van Diest PJ, Juergens H (2007). Epidermal growth factor receptor expression in high-grade osteosarcomas is associated with a good clinical outcome. Clin Cancer Res.

[29] Kersting C, Agelopoulos K, Schmidt H, Korsching E, August C, Gosheger G (2008). Biological importance of a polymorphic CA sequence within intron 1 of the epidermal growth factor receptor gene (EGFR) in high grade central osteosarcomas. Genes Chromosomes Cancer.

[30] Pahl JH, Kwappenberg KM, Varypataki EM, Santos SJ, Kuijjer ML, Mohamed S (2014). Macrophages inhibit human osteosarcoma cell growth after activation with the bacterial cell wall derivative liposomal muramyl tripeptide in combination with interferon-gamma. J Exp Clin Cancer Res.

[31] Gobin B, Moriceau G, Ory B, Charrier C, Brion R, Blanchard F (2014). Imatinib mesylate exerts anti-proliferative effects on osteosarcoma cells and inhibits the tumour growth in immunocompetent murine models. PLoS One.

[32] Messerschmitt PJ, Rettew AN, Brookover RE, Garcia RM, Getty PJ, Greenfield EM (2008). Specific tyrosine kinase inhibitors regulate human osteosarcoma cells in vitro. Clin Orthop Relat Res.

[33] Hughes DP, Thomas DG, Giordano TJ, McDonagh KT, Baker LH (2006). Essential erbB family phosphorylation in osteosarcoma as a target for CI-1033 inhibition. Pediatr Blood Cancer.

[34] Pahl JH, Ruslan SE, Buddingh EP, Santos SJ, Szuhai K, Serra M (2012). Anti-EGFR antibody cetuximab enhances the cytolytic activity of natural killer cells toward osteosarcoma. Clin Cancer Res.

[35] Hou CH, Lin FL, Tong KB, Hou SM, Liu JF (2014). Transforming growth factor alpha promotes osteosarcoma metastasis by ICAM-1 and PI3K/Akt signaling pathway. Biochem Pharmacol.

[36] Andreopoulou E, Vigoda IS, Valero V, Hershman DL, Raptis G, Vahdat LT (2013). Phase I-II study of the farnesyl transferase inhibitor tipifarnib plus sequential weekly paclitaxel and doxorubicin-cyclophosphamide in HER2/neu-negative inflammatory carcinoma and non-inflammatory estrogen receptor-positive breast carcinoma. Breast Cancer Res Treat.

[37] Teoh D, Secord AA (2012). Antiangiogenic agents in combination with chemotherapy for the treatment of epithelial ovarian cancer. Int J Gynecol Cancer.

[38] Gross M, Higano C, Pantuck A, Castellanos O, Green E, Nguyen K (2007). A phase II trial of docetaxel and erlotinib as first-line therapy for elderly patients with androgen-independent prostate cancer. BMC Cancer.

[39] Agarwal S, Sane R, Gallardo JL, Ohlfest JR, Elmquist WF (2010). Distribution of gefitinib to the brain is limited by P-glycoprotein (ABCB1) and breast cancer resistance protein (ABCG2)-mediated active efflux. J Pharmacol Exp Ther.

[40] Oda Y, Matsumoto Y, Harimaya K, Iwamoto Y, Tsuneyoshi M (2000). Establishment of new multidrug-resistant human osteosarcoma cell lines. Oncol Rep.

[41] Milano G, Spano JP, Leyland-Jones B (2008). EGFR-targeting drugs in combination with cytotoxic agents: from bench to bedside, a contrasted reality. Br J Cancer.

[42] Sirotnak FM, Zakowski MF, Miller VA, Scher HI, Kris MG (2000). Efficacy of cytotoxic agents against human tumor xenografts is markedly enhanced by coadministration of ZD1839 (Iressa), an inhibitor of EGFR tyrosine kinase. Clin Cancer Res.

[43] Cunningham D, Humblet Y, Siena S, Khayat D, Bleiberg H, Santoro A (2004). Cetuximab monotherapy and cetuximab plus irinotecan in irinotecan-refractory metastatic colorectal cancer. N Engl J Med.

[44] Ren W, Korchin B, Zhu QS, Wei C, Dicker A, Heymach J (2008). Epidermal growth factor receptor blockade in combination with conventional chemotherapy inhibits soft tissue sarcoma cell growth in vitro and in vivo. Clin Cancer Res.

[45] van Cruijsen H, Voest EE, Punt CJ, Hoekman K, Witteveen PO, Meijerink MR (2010). Phase I evaluation of cediranib, a selective VEGFR signalling inhibitor, in combination with gefitinib in patients with advanced tumours. Eur J Cancer.

[46] Jakacki RI, Hamilton M, Gilbertson RJ, Blaney SM, Tersak J, Krailo MD (2008). Pediatric phase I and pharmacokinetic study of erlotinib followed by the combination of erlotinib and temozolomide: a Children’s Oncology Group Phase I Consortium Study. J Clin Oncol.

