# Supplementary material for: EGFR is not a major driver for osteosarcoma cell growth in vitro but contributes to starvation and chemotherapy resistance
Source: J Exp Clin Cancer Res. 2015 Nov 2;34:134. doi: 10.1186/s13046-015-0251-5 (PMC4630894; doi:10.1186/s13046-015-0251-5)
Supplement: Additional file 1: Table S1. — Phenotypic characteristics as well as EGFR and ABCB1 gene expression of the investigated osteosarcoma cell models. (DOCX 14 kb) [file 13046_2015_251_MOESM1_ESM.docx]

**Additional file 1: Table S1.** Phenotypic characteristics as well as EGFR and ABCB1 gene expression of the investigated osteosarcoma cell models

| Cell line | Subtype/Differentiation | Culture medium^3^ | EGFR protein^4^ | EGFR mRNA^5^ | ABCB1  mRNA^6^ |  |
| --- | --- | --- | --- | --- | --- | --- |
| MG-63 | fibroblastic^1^ | RPMI | ++ | 0.5 | 0.01 |  |
| HOS/CRL1543 | mixed^1^ | RPMI | +++ | 0.4 | 0.09 |  |
| Saos-2 | epithelial^1^ | RPMI | +/- | 0.2 | 0.22 |  |
| U-2 OS | epithelial^1^ | IMDM | ++ | 0.1 | 1.85 |  |
| OS-10 | fibroblastic^2^ | IMDM | +++ | 0.5 | 0.75 |  |
| OS-9 | osteoblastic^2^ | IMDM | + | 0.2 | 0.63 |  |
| SARG | osteoblastic^2^ | IMDM | + | 0.1 | 0.43 |  |
| IOR-MOS | osteoblastic^2^ | IMDM | +++ | 0.6 | 0.10 |  |
| HL-NG | fibroblastic | RPMI | +/- | 0.3 | n.d.^7^ |  |

^1^ according to ATCC

^2^ according to Mohseny et al.

^3^ RPMI= RPMI–1640 medium; IMDM= Iscove's Modified Dulbecco's Medium

^4^ from Western blot analyses in Fig. 1A

^5^ relative to EGFR overexpressing Calu-3 lung cancer cells

^6^ relative to the moderately ABCB1-overexpression triapine-resistant SW480 subline SW480/Tria

^7^ not detected
