# Supplementary figures and images for: EGFR is not a major driver for osteosarcoma cell growth in vitro but contributes to starvation and chemotherapy resistance
Source: J Exp Clin Cancer Res. 2015 Nov 2;34:134. doi: 10.1186/s13046-015-0251-5 (PMC4630894; doi:10.1186/s13046-015-0251-5)

EGFR

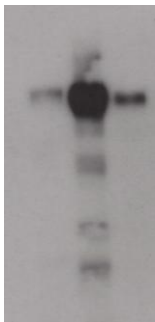

HL-NG  
U-2 OS  
Saos-2

Supplement: Additional file 2: Figure S1. — EGFR expression in osteosarcoma cells. For lowly EGFR positive osteosarcoma cell lines in Fig. 1a Western blots of membrane-enriched fractions were prepared and subjected to extended film exposure. (PDF 26 kb) [file 13046_2015_251_MOESM2_ESM.pdf]

**A**

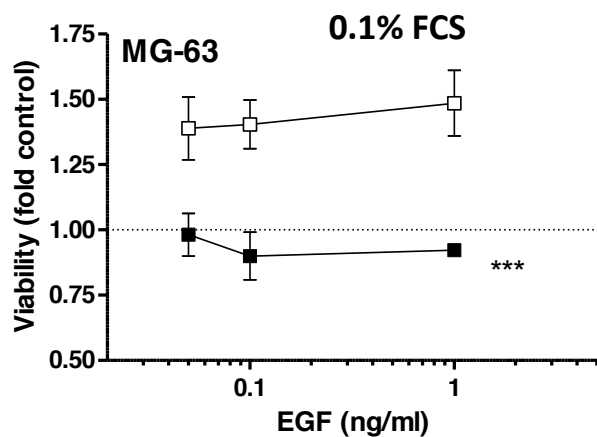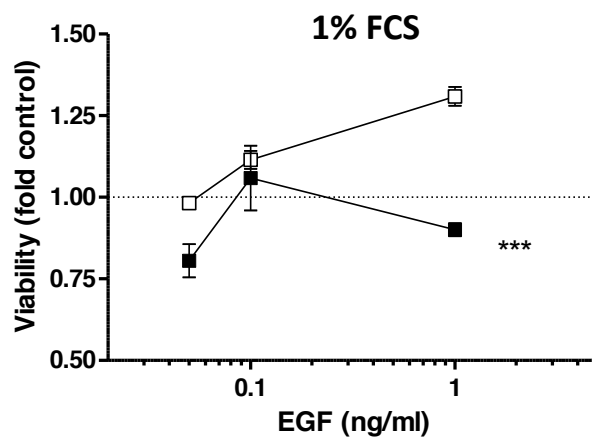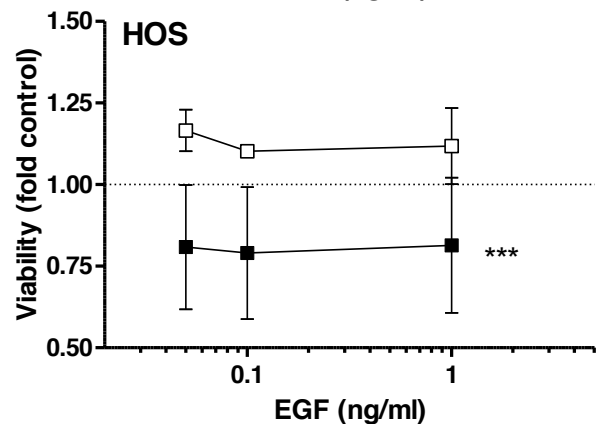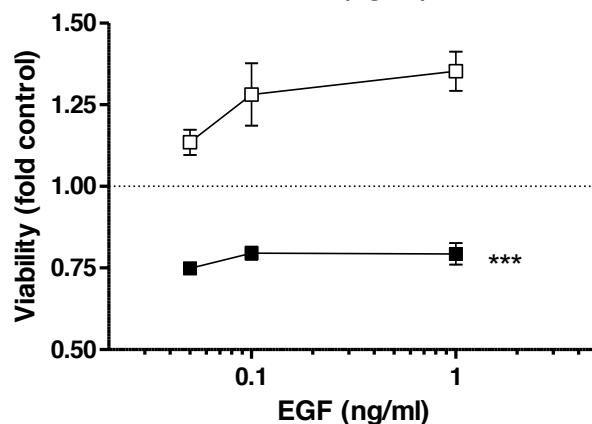

**B**

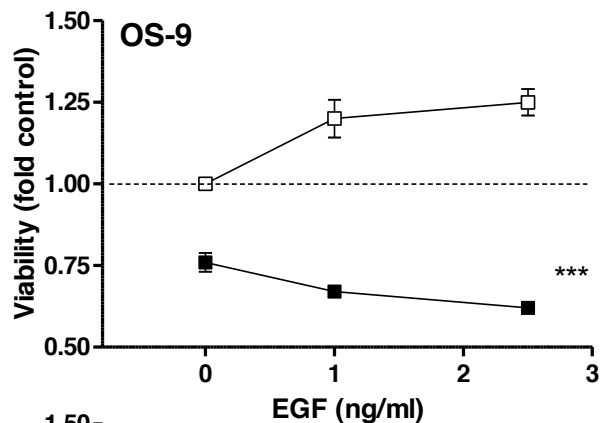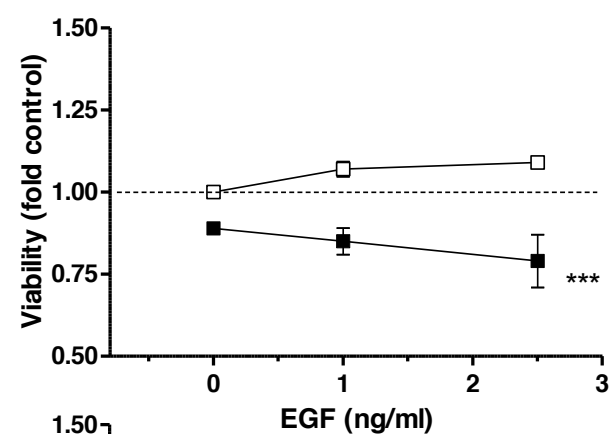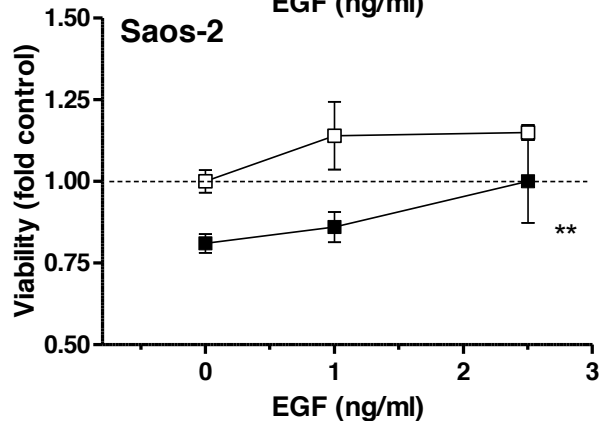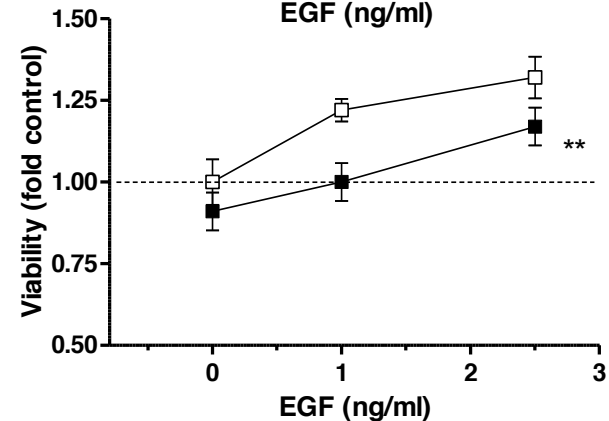

□ Gefitinib 0  $\mu$ M  
■ Gefitinib 5  $\mu$ M

Supplement: Additional file 3: Figure S2. — Impact of EGF and EGFR inhibition on starvation survival of osteosarcoma cells with comparably high (A) and low (B) EGFR expression levels. Viability of osteosarcoma cells was determined by MTT assays after 72 h serum starvation (1 % or 0.1 % FCS) under increasing EGF concentrations without or with gefitinib (5 μM) as indicated. Significance of the gefitinib impact: ** p < 0.01; *** p < 0.001 by Two-way ANOVA with Bonferroni’s post hoc test. (PDF 71 kb) [file 13046_2015_251_MOESM3_ESM.pdf]

Sevelda et al. Fig. S-3

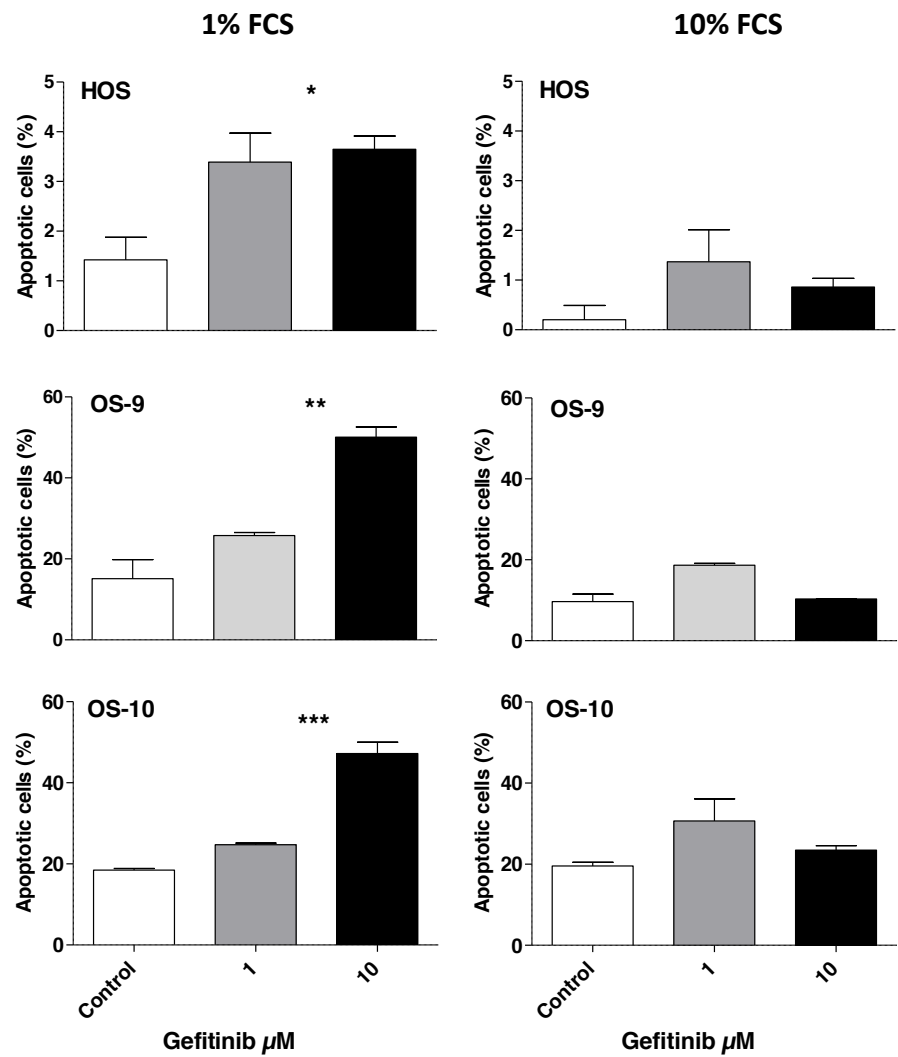

Supplement: Additional file 4: Figure S3. — Impact of serum starvation on apoptosis induction by gefitinib in osteosarcoma cells. The indicated osteosarcoma cell lines were treated with gefitinib (1 μM and 10 μM) for 24 h in 1 and 10 % FCS containing culture medium. Hoechst 33258 and propidium iodide were added for 4 h and photomicrographs of 4 optical fields per well were evaluated for cells with condensed chromatin indicating apoptosis execution. * p < 0.05; ** p < 0.01; *** p < 0.001 by One-way ANOVA with Bonferroni’s post hoc test. (PDF 62 kb) [file 13046_2015_251_MOESM4_ESM.pdf]

Sevelda et al. Fig. S-4

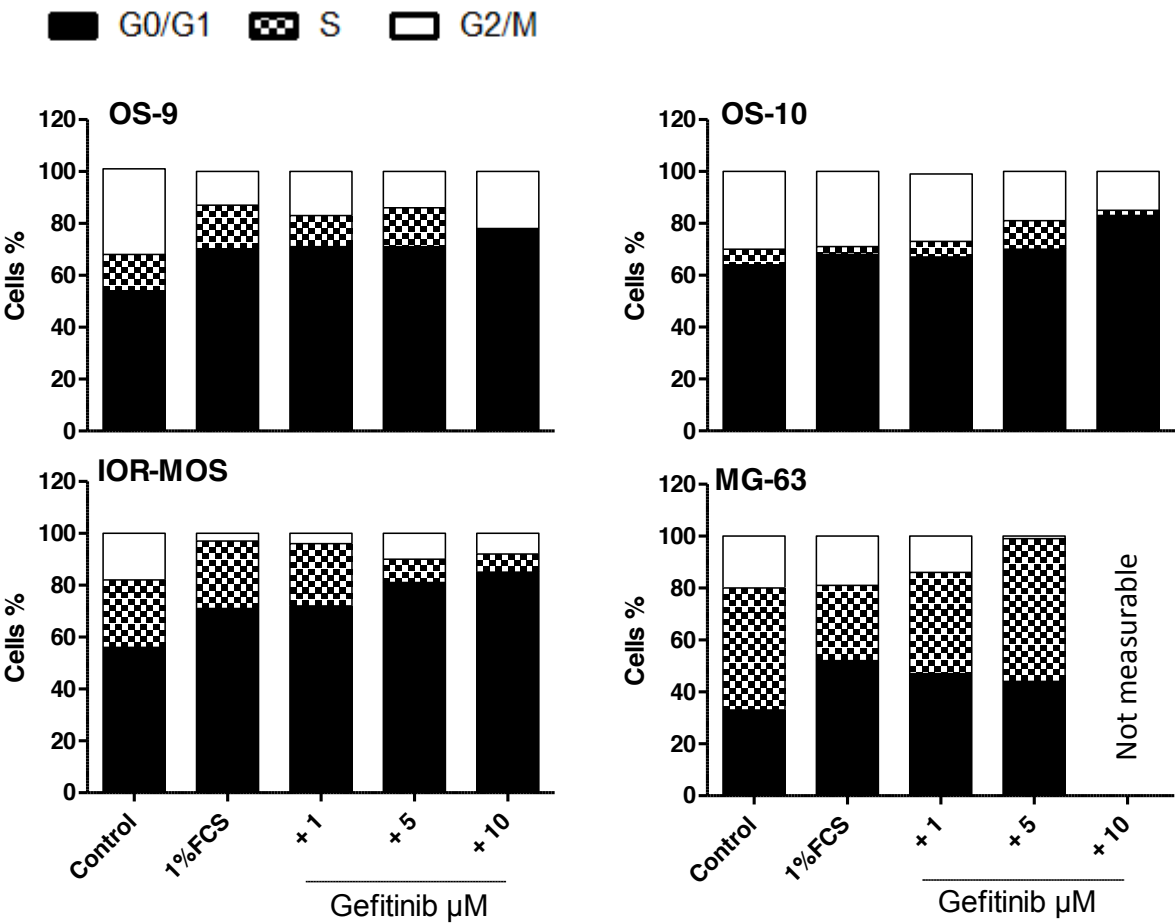

Supplement: Additional file 5: Figure S4. — Impact of EGFR inhibition by gefitinib on cell cycle distribution. The indicated osteosarcoma cell lines were treated with gefitinib (1 μM, 5 μM and 10 μM) for 24 h in 1 % FCS containing culture medium and opposed to untreated cells at 10 % FCS (control). Cell cycle distribution was analysed by PI staining followed by FACS analysis. (PDF 56 kb) [file 13046_2015_251_MOESM5_ESM.pdf]
